# Anatomical, histological, and scanning electron microscopic features of the esophagus and crop in young and adult domestic pigeons (*Columba livia Domestica*)

**DOI:** 10.1186/s12917-024-04147-z

**Published:** 2024-09-23

**Authors:** Mohamed A. A. Mahdy, Elsayed S. I. Mohammed

**Affiliations:** 1 Department of Anatomy and Histology, Faculty of Veterinary Medicine, King Salman International University, Ras Sudr, Egypt; 2 Department of Anatomy and Embryology, Faculty of Veterinary Medicine, South Valley University, Qena, Egypt; 3 Department of Histology and Cytology, Faculty of Veterinary Medicine, South Valley University, Qena, 83523 Egypt; 4 Avian Research Center, King Faisal University, Al-Ahsa, 31982 Saudi Arabia

**Keywords:** Crop, Esophagus, Esophageal glands, Pigeon

## Abstract

**Background:**

Pigeons (*Columba livia*) are mainly raised as a source of animal protein, racing, leisure and as an experimental animal. The present study investigated the morphology of the esophagus in the young and adult domestic pigeon, *Columba livia domestica.*

**Methods:**

Ten young and ten adult, normal, and healthy pigeons were collected from the local breeders. Samples from different parts of esophagus and crop were examined grossly, by stereomicroscopy, scanning and light microscopy.

**Results:**

The esophagus consisted of a long cervical part, a crop, and a short thoracic part. The crop was represented by a thin-walled outpouching with two lateral diverticula. The mucosa presented wavy fine folds in the cervical esophagus, irregular folds in the lateral diverticula giving it a corrugated appearance, and prominent longitudinal folds with several gland openings in the middle and lower parts of the crop, as well as in the thoracic esophagus. The density of gland openings was higher in adult pigeons than that in young pigeons. The mucosa of the esophagus was lined by non-keratinized stratified squamous epithelium. The shape, height, and branching of the mucosal folds differed between young and adult pigeons. Mucous-secreting alveoli were detected in the middle part of the crop as well as in the thoracic esophagus, but not in the cervical esophagus or lateral diverticula of the crop.

**Conclusion:**

The variations between the young and adult pigeons suggest a functional adaptation of adult pigeons to their diet compared to young pigeons.

## Introduction

Pigeons (*Columba livia*) are mainly raised as a source of animal protein [1], ornamental birds, experimental animals [2, 3], and a model to study the evolutionary genetics of avian and vertebrate species [4]. In developing countries, pigeons are raised mainly for squabs production which are characterized by their rapid growth [5], high nutritive value, and low cholesterol content [1].

The avian esophagus is a muscular tube that connects the oropharyngeal cavity to the glandular stomach [6, 7]. It is divided mainly into cervical and thoracic parts [6]. The avian crop is an outpouching of the esophagus just before entering the thoracic cavity [8, 9]. The size and shape of the crop are species-specific characteristics depending on the diet [10]. The crop temporarily stores the ingested food. Additionally, the crop plays a role in moistening food to support enzymatic degradation in the stomach [10, 11]. Furthermore, the crops of both male and female pigeons and doves secrete crop milk under the effect of the prolactin hormone during the brooding period to feed their squabs [10, 12, 13].

The gross morphology of the esophagus and crop has been studied in different avian species, including wood pigeons [14], house sparrows [15], and partridges [16]. Moreover, chicken crops have been studied using scanning electron microscopy (SEM) [17]. Furthermore, the microscopic structure of the esophagus has been described in numerous bird species, including rock pigeons [7], Japanese quails [18], chickens [19], and turkeys [20]. Variations in the histomorphometric measurements of the esophagus have been reported in adult male and female homing pigeon [21]. Recently, species-specific variations have been reported in the histologic structure of the esophagus and crops of pigeons, cattle egrets, and ducks representing granivorous, carnivores, and omnivorous birds, respectively [6, 22]. The authors referred to these variations as differences in the dietary habits between these species.

Trichomonosis and candidiasis are the most common diseases affecting the upper digestive system of a wide range of avian species especially Columbiformes causing important medical and commercial implications [23, 24]. Trichomonosis is a protozoan disease with a prevalence rate ranging from 35.1 to 75% in pigeons [25, 26]. *Trichomonas gallinae* induces necrotic lesions in the oropharynx, esophagus, and crop of pigeons resulting in a high mortality rate in young pigeons due to starvation and suffocation [23, 27, 28]. While candidiasis is a fungal disease with a prevalence rate ranging from 31.7 to 41.2% in pigeons [24, 29]. Candidiasis is characterized by formation of white plaques on the oropharyngeal mucosa causing crop stasis, regurgitation, and weight loss [24]. To accurately interpret the histopathological changes induced by trichomonosis infection, thorough knowledge of the morphological structure of the normal esophagus and crop of pigeon is required. Therefore, the current study was conducted to investigate the morphology of the esophagus in young and adult pigeons (*Columba livia domestica)* using gross observation, stereomicroscopy, light microscopy, and scanning electron microscopy.

## Methods

### Sample collection

Ten young pigeons (squabs, 3 weeks old), weighting 270–320 g body weight, and 10 adult pigeons (18 months old), weighting 345–425 g body weight, were purchased from local breeders in Qena Governorate, Egypt. The pigeons were normal and apparently healthy with no signs of disease. The birds were euthanized by using carbon dioxide (CO_2_) inhalation [30]. Birds were placed in a chamber with 100% CO_2_ concentration. This method induced rapid loss of consciousness followed by death. All procedures were performed in accordance with the ARRIVE guidelines and the guidelines of the Institutional Ethical Committee of Faculty of Veterinary Medicine, South Valley University, Egypt (Approval number:17b-07-2021).

### Gross anatomy and stereomicroscopy

Seven birds of each age were used for the gross anatomy. The esophagus and crop were exposed and photographed *in-situ*. The esophagus and crop were then removed from the body and washed with saline, and the lumen was cut longitudinally. Gross photographs were obtained using a Nikon COOLPIX S9400 digital camera. The internal surface was examined using a stereomicroscope (SZ61, Olympus, Tokyo, Japan) and photographed using a digital camera (XCAM, ToupTek, Zhejiang, China).

### Scanning electron microscopy

Tissue samples were collected from three birds of each age group. Samples from the cervical and thoracic esophagus, lateral diverticulum, and middle part of the crop were flushed with normal saline and fixed in 2.5% paraformaldehyde and 2.5% glutaraldehyde solution in 0.1 M phosphate buffer (pH 7.4) for 24 h at 4^o^C. The specimens were post-fixed in 1% osmium tetroxide, dehydrated in a graded alcohol series, and critically point-dried in liquid carbon dioxide. Samples were coated with gold using a SPI-Module Sputter Coater and examined under a scanning electron microscope (JSM-5500 LV, Joel, Japan) operated at 10–20 kV [7, 31] in the central lab of South Valley University, Egypt.

### Histological and histochemical analysis

Samples were taken from four different areas as follows: the cervical and thoracic parts of the esophagus approximately 1 cm away from the crop, the longitudinal ridges in the middle part of the crop, and the lateral sacs of the crop. The samples were flushed with normal saline and fixed in 10% neutral buffered formalin [32]. The tissue samples were processed routinely for paraffin embedding. Serial Sections (4 μm thick) were cut using an automated rotatory microtome (Leica RM2235, Leica Biosystems, Wetzlar, Germany) and stained with Mayer’s hematoxylin and eosin (HE) for general histological description, and Crossman’s trichrome for connective tissue staining. Histochemical staining was performed using the periodic acid–Schiff (PAS) technique to detect neutral mucopolysaccharides, the Alcian blue technique to demonstrate acidic mucopolysaccharides, and the Alcian blue-periodic acid-Schiff (AB-PAS) technique [33]. Sections were examined and photographed using a Leica DMLS microscope (Leica Microsystems) equipped with a Leica ICC50 HD camera.

### Histomorphometric measurements and statistical analysis

Histomorphometry was performed on HE-stained sections (four representative non-overlapping fields/bird, and three birds per age were used) using Image-J 1.46r software (National Institute of Health, Bethesda, Maryland, USA). Histomorphometric measurements included the length and width of the mucosal folds [7]. Normal distribution of the data was assessed by Shapro-Wilk test. Measurements were analyzed by Student’s *t*-test using the IBM SPSS software v22 (Chicago, IL, USA). Data were expressed as Mean ± SD.

## Results

### Gross anatomy

The esophagus of the pigeons was represented by a long tube connecting the pharynx to the proventriculus. It could be divided into three parts: the cervical part, crop, and thoracic part.

#### The cervical esophagus

**Fig. 1:**
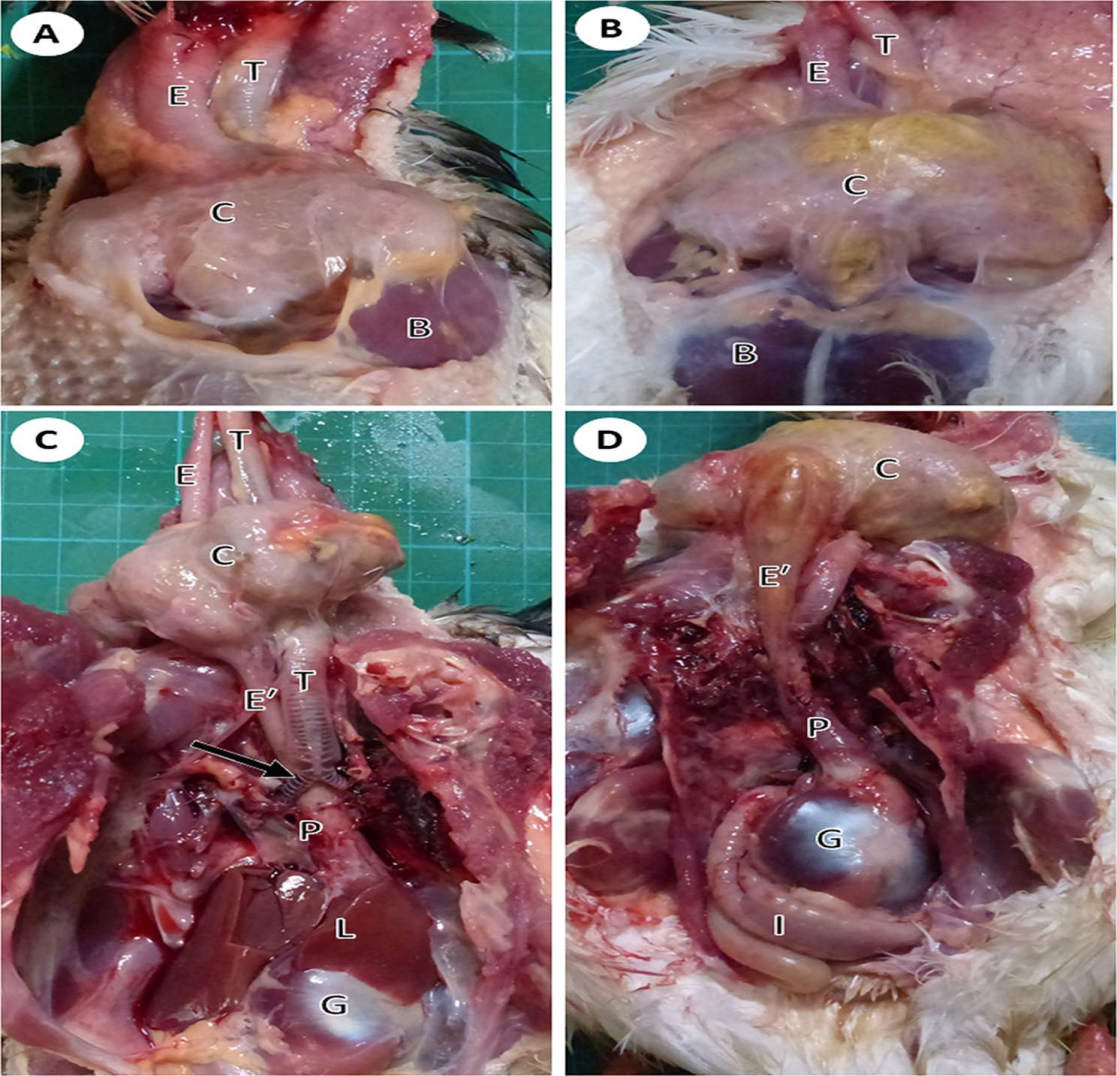
A gross micrograph showing the course and relationship of the esophagus in situ in young (**A**, **C**) and adult (**B**, **D**) pigeons. (**A**, **B**) The cervical esophagus (E) was located on the right side of the trachea (T), the crop (C) was located at the entrance to the thoracic cavity in front of the breast muscle (B). (**C**, **D**) The thoracic esophagus (Eʹ) after removal of the heart (H) and liver (L) lied dorsal to the trachea and the syrinx (Arrow), the proventriculus (P), the gizzard (G), and the intestine (I)

**Fig. 2:**
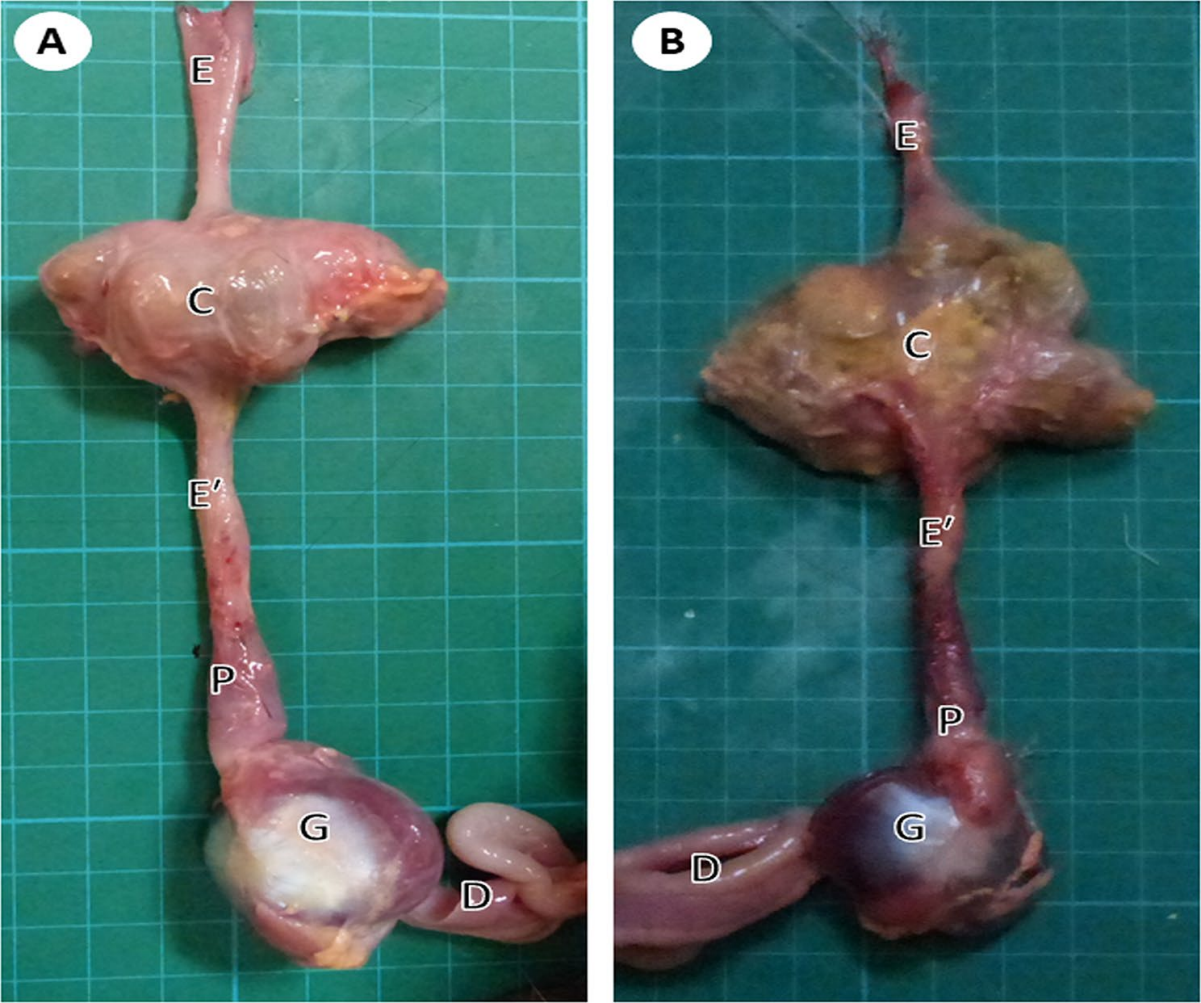
A gross micrograph of the isolated esophagus of young (**A**) and adult (**B**) pigeons showing the cervical esophagus (E), crop (C), thoracic esophagus (Eʹ), proventriculus (P), gizzard (G), and duodenum (D)

The cervical part was represented by a thin-walled long tube that extended from the pharyngoesophageal junction to the entrance of the thoracic cavity, where it enlarged to form the crop (Fig. 1). This part lied in the midline dorsal to the larynx and trachea in the cranial cervical region. In the middle cervical region, the esophagus and trachea were displaced on both sides of the neck and the esophagus extended along the right ventrolateral side of the neck. Esophageal and tracheal displacements were more obvious in adult pigeons (Fig. 1). Stereomicroscopic observations of the internal surface revealed the presence of fine longitudinal folds (Fig. 2).

#### The crop

**Fig. 3:**
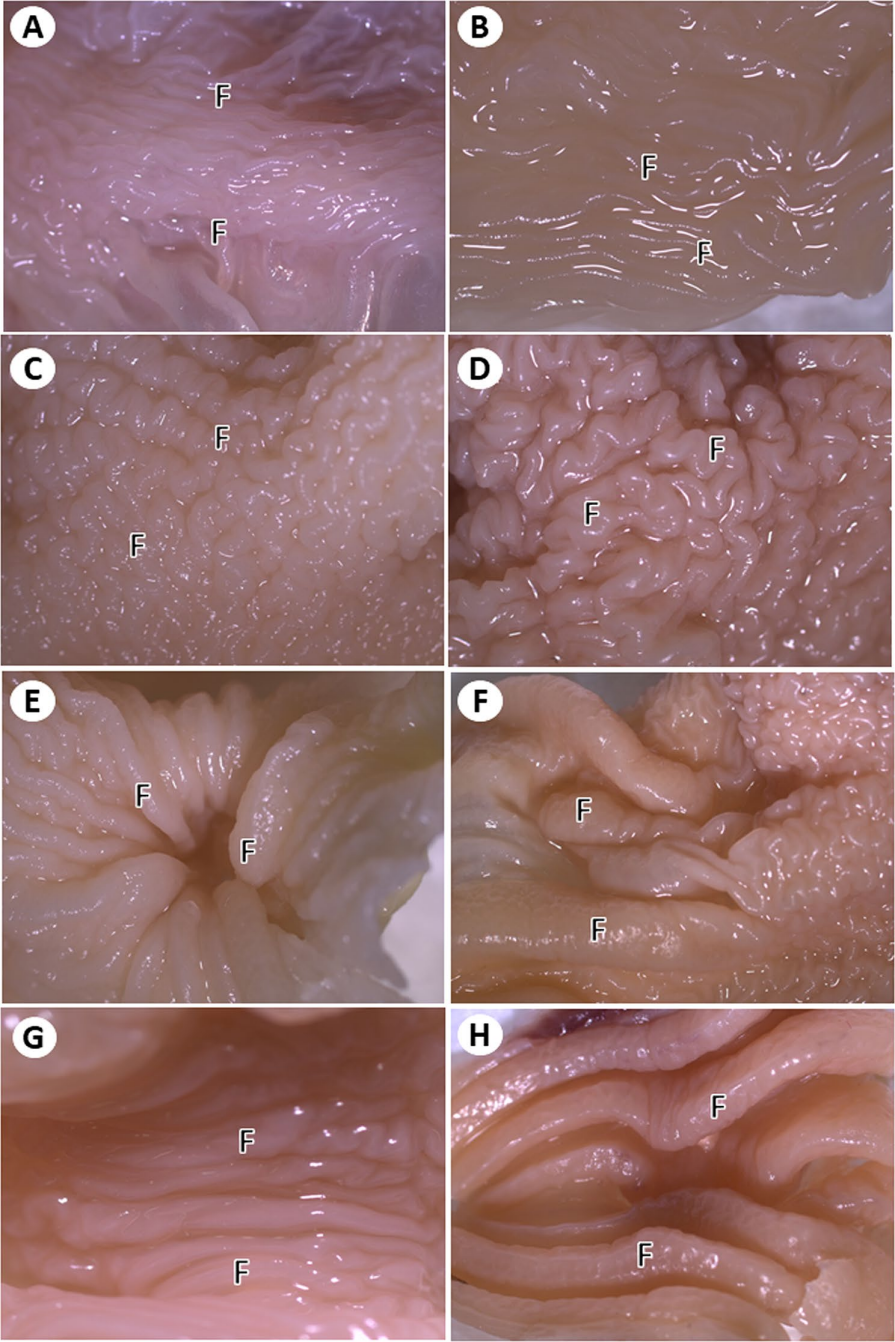
Stereomicroscopic images of the cervical esophagus (**A**, **B**), lateral diverticulum of the crop (**C**, **D**), central and lower parts of the crop (**E**, **F**), and thoracic part of the esophagus (**G**, **H**) in young (**A**, **C**, **E**, **G**) and adult (**B**, **D**, **F**, **H**) pigeons showing different mucosal folds (F)

The crop was represented by a thin-walled outpouching with two lateral diverticula (Fig. 2). The inner surface of the crop, as shown by stereomicroscopy, was characterized by the presence of irregular folds in the lateral diverticula, giving it a corrugated appearance. While the middle and lower parts of the crop showed 4–5 very prominent longitudinal folds separated by a few less prominent folds, these folds were separated by deep grooves (Fig. 3).

#### The thoracic *esophagus*

The thoracic part of the esophagus was shorter than the cervical part. It was represented by a thick-walled narrow tube that extended from the crop to the junction with the proventriculus (Fig. 2). It lied in the thoracoabdominal cavity dorsal to the trachea, syrinx, and base of the heart (Fig. 1). Stereomicroscopic observation of the internal surface revealed prominent longitudinal folds separated by deep grooves (Fig. 3).

### Scanning electron microscopy

#### The *cervical* esophagus

**Fig. 4:**
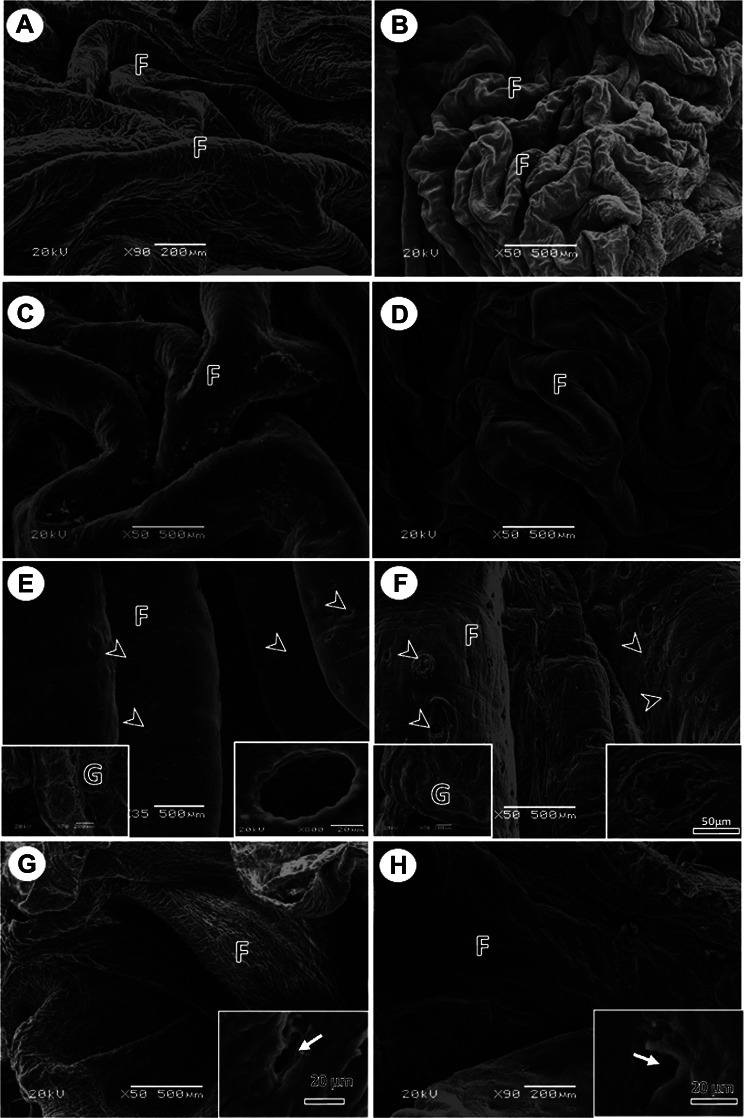
SEM micrographs of the cervical of the upper part of the esophagus (**A**, **B**), lateral diverticulum of the crop (**C**, **D**), central and lower parts of the crop (**E**, **F**), and thoracic part of the esophagus (**G**, **H**) in young (**A**, **C**, **E**, **G**) and adult (**B**, **D**, **F**, **H**) pigeons. The mucosal folds (F), openings of the esophageal glands (arrowheads). Insets in (**E**, **F**): the right one showed the cut surface with numerous abundant lobules of esophageal glands (G), while the left one showed an opening of the esophageal gland. Insets in (**G**, **H**) showed opening of the esophageal gland (arrow)

The mucosa of the cervical esophagus was folded in both young and adult pigeons. The mucosal folds were wavy and fine, separated by fine grooves in young pigeons, and highly folded in adult pigeons compared with young pigeons. The mucosal folds had fine microridges (Fig. 4).

#### The *crop*

The mucosa of the lateral diverticula of the crop was characterized by the presence of irregularly branched folds separated by grooves in both young and adult pigeons. The number of branched folds was higher in adult pigeons than in the young pigeons. The folds carried desquamated flakes (Fig. 4). The mucosal folds in the middle and lower parts of the crop were very prominent, straight, and separated by deep grooves. In young pigeons, the longitudinal folds appeared smooth and had several rounded to oval openings of the esophageal glands. In adult pigeons, the longitudinal mucosal folds appeared rough owing to the presence of microridges. They had several esophageal gland openings. The density of gland openings was higher in adult pigeons than that in young pigeons. Some gland openings appeared as rounded or oval invaginations of the mucosa. Some gland openings were surrounded by flat, superficial cells that underwent exfoliation (Fig. 4). The cut surface showed numerous lobules of the esophageal glands in both young and adult pigeons.

#### The thoracic *esophagus*

The mucosal folds of the thoracic esophagus were prominent, straight, and separated by deep grooves. The folds were thicker and higher in adult pigeons than in young pigeons, and their surfaces appeared rough due to the presence of microridges. They had openings of esophageal glands, but the frequency of gland openings was less than that present in the middle part of the crop (Fig. 4).

### Light microscopy

#### The cervical *esophagus*

**Fig. 5:**
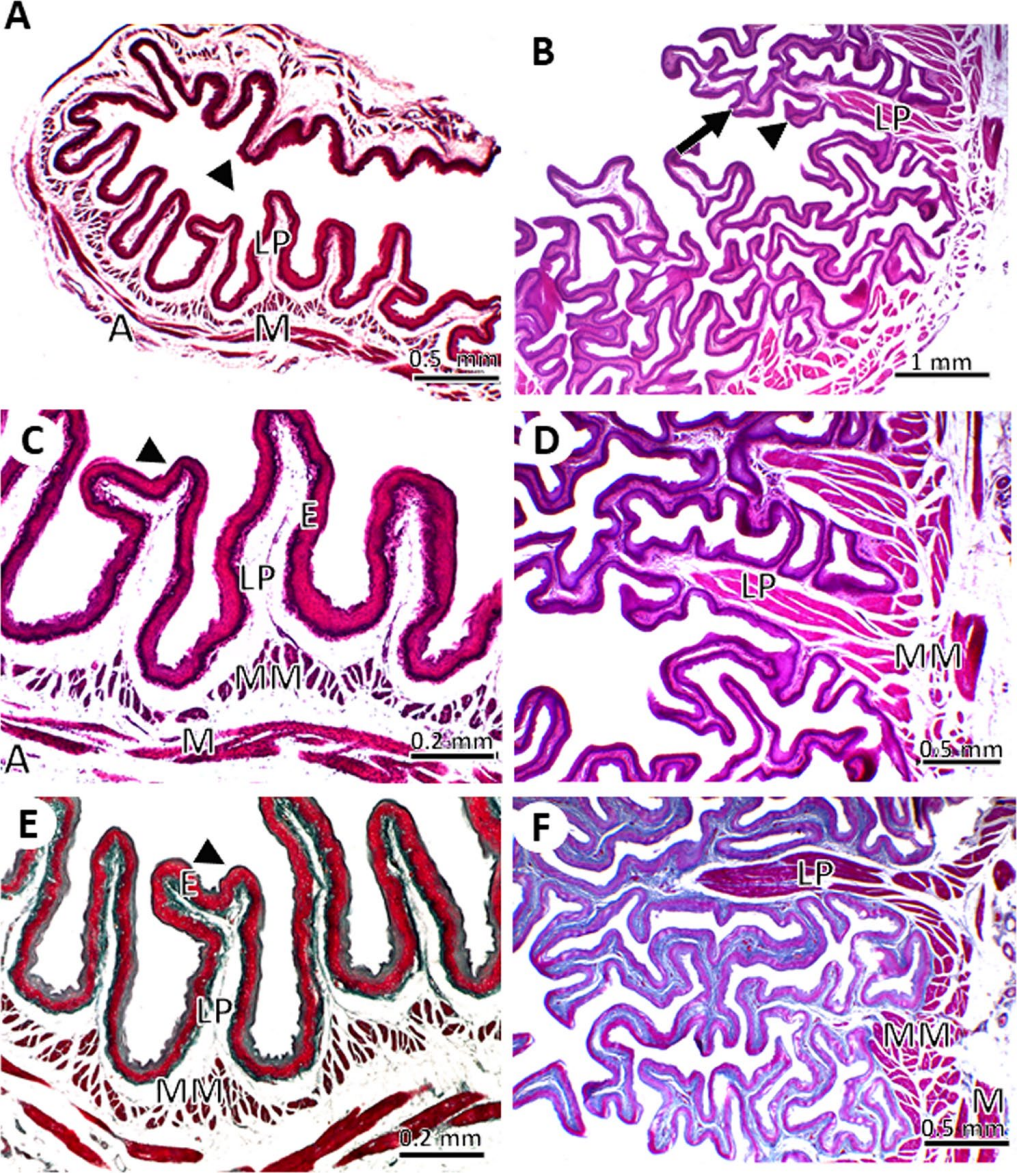
Light micrographs of the cervical part esophagus in young (**A**, **C**, **E**) and adult (**B**, **D**, **F**) pigeons. The mucosal folds of the cervical esophagus lined by non-keratinized stratified squamous epithelium (**E**), lamina propria (LP), muscularis mucosa (MM), muscularis (M) and adventitia (A). Note the secondary mucosal folds (arrowhead), and tertiary mucosal folds (black arrows) that arise from the primary fold. (**A**-**D**) stained with HE stain, (**E**, **F**) stained with Crossman’s trichrome stain

The wall of the cervical esophagus, crop, and thoracic esophagus consisted of four tunics in both young and adult pigeons: tunica mucosa, tunica submucosa, tunica muscularis, and tunica adventitia (for the cervical part) or tunica serosa (for the thoracic part). The tunica mucosa was lined with non-keratinized stratified squamous epithelium. The lamina propria was composed of sparse collagenous connective tissue in young pigeons and thick collagenous connective tissue with several blood vessels, lymph vessels, and immune cells in adult pigeons. The tunica submucosa consisted of loose connective tissue. The tunica muscularis was composed of inner circular and outer longitudinal layers and was surrounded by either thin tunica adventitia in the cervical esophagus or tunica serosa in the crop and thoracic esophagus (Fig. 5).

The mucosa was thrown into mucosal folds. The shapes of these folds differed in the different parts of the esophagus and between adult and young pigeons. The cervical esophagus in young pigeons had primary mucosal folds with very few secondary folds, whereas in adult pigeons it was highly folded, with primary, secondary, and tertiary folds. The height of the mucosal folds was significantly greater in adults than in young pigeons (3.74 ± 0.96 *Vs*. 0.92 ± 0.28 mm, *P* < 0.05). In young pigeons, the mucosal folds were devoid of muscles from the muscularis mucosa. However, in adult pigeons, the muscularis mucosa was more developed than that in young pigeons and formed a longitudinal layer of smooth muscle fibers that sent a circular bundle into the primary mucosal folds (Fig. 5).

#### The *crop*

**Fig. 6:**
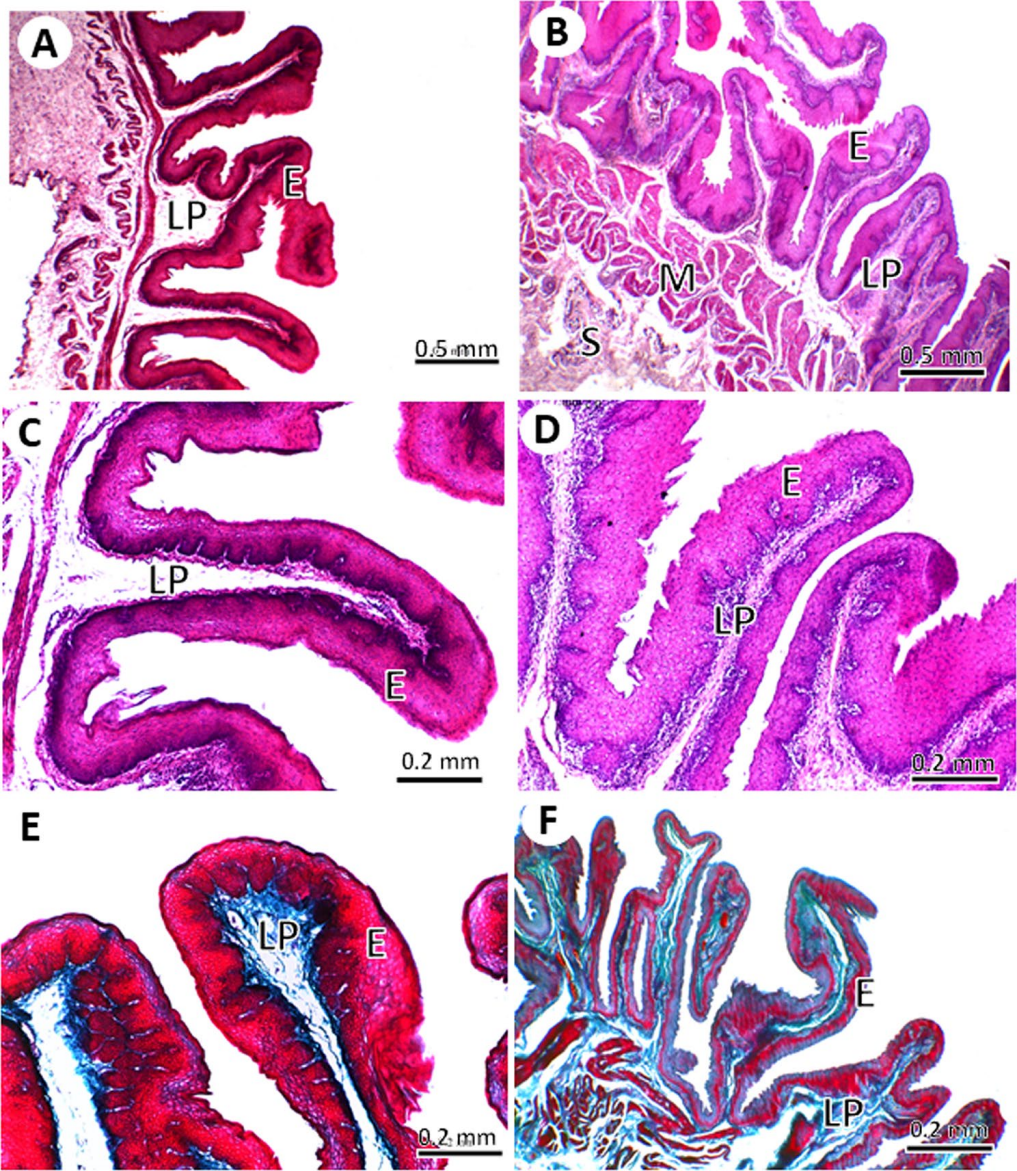
Light micrographs of the lateral diverticulum of the crop in young (**A**, **C**, **E**) and adult (**B**, **D**, **F**) pigeons. The mucosal folds were lined by non-keratinized stratified squamous epithelium (**E**), lamina propria (LP), muscularis (M) and serosa (S). (**A**-**D**) stained with HE stain, (**E**, **F**) stained with Crossman’s trichrome stain

**Fig. 7:**
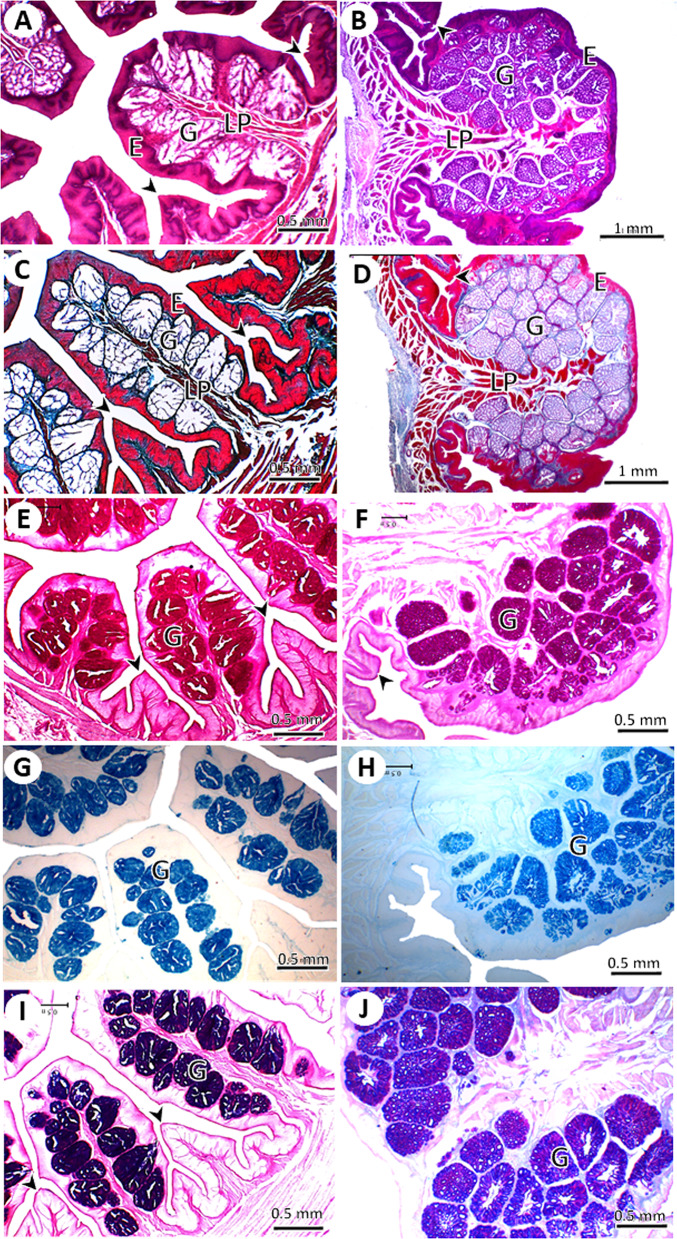
Light micrographs of the central and lower parts of the crop in young (**A**, **C**, **E**, **G**, **I**) and adult (**B**, **D**, **F**, **H**, **J**) pigeons. The mucosal folds were lined by non-keratinized stratified squamous epithelium (**E**), lamina propria (LP) and gland (G). (A, B) stained with HE stain; (**C**, **D**) stained with Crossman’s trichrome stain, (**E**, **F**) stained with PAS technique, (**G**, **H**) stained with Alcian blue; and (**I**, **J**) stained with Alcian blue-PAS technique

The mucosa of the lateral diverticula of the crop formed primary folds with a few secondary folds, in both young and adult pigeons. The folds were significantly higher (1.05 ± 0.19 *Vs*. 0.89 ± 0.1 mm, *P* = 0.03) in adult pigeons than in young pigeons with no significant difference in fold width between the two age groups (0.41 ± 0.07 *Vs*. 0.35 ± 0.06 mm, *P =* 0.01). The muscularis mucosa was thicker and more well-developed in adults than in young pigeons (Fig. 6). The mucosa of the middle and lower parts of the crop formed primary mucosal folds separated by several secondary folds. The shape of the primary mucosal folds was leaf-like in young pigeons, but club-like in adult pigeons. The mucosa of the middle and lower parts of the crop formed primary mucosal folds separated by several secondary folds. The shape of the primary mucosal folds was leaf-like in young pigeons, but club-like in adult pigeons. The folds were significantly higher (2.86 ± 0.16 *Vs*. 1.99 ± 0.45 mm, *P* = 0.00) and in adult pigeons than in young pigeons. Moreover, the folds were significantly wider in adult pigeons than in young pigeons (3.30 ± 0.33 *Vs*. 1.12 ± 0.22 mm, *P* = 0.02). The primary folds contained numerous mucous-secreting alveoli, were positive for PAS stain, were separated by connective tissue trabeculae, and had a circular bundle of smooth muscles that arose from the longitudinal smooth muscles of the muscularis mucosa. The gland secretions included acidic and neutral mucopolysaccharides, which were positive for AB-PAS staining. The larger secondary folds of young pigeons may contain smooth muscles originating from the muscularis mucosa with no glands (Fig. 7). The number and branching of mucous glands were higher in the adult pigeons than in the young pigeons.

#### The thoracic *esophagus*

**Fig. 8:**
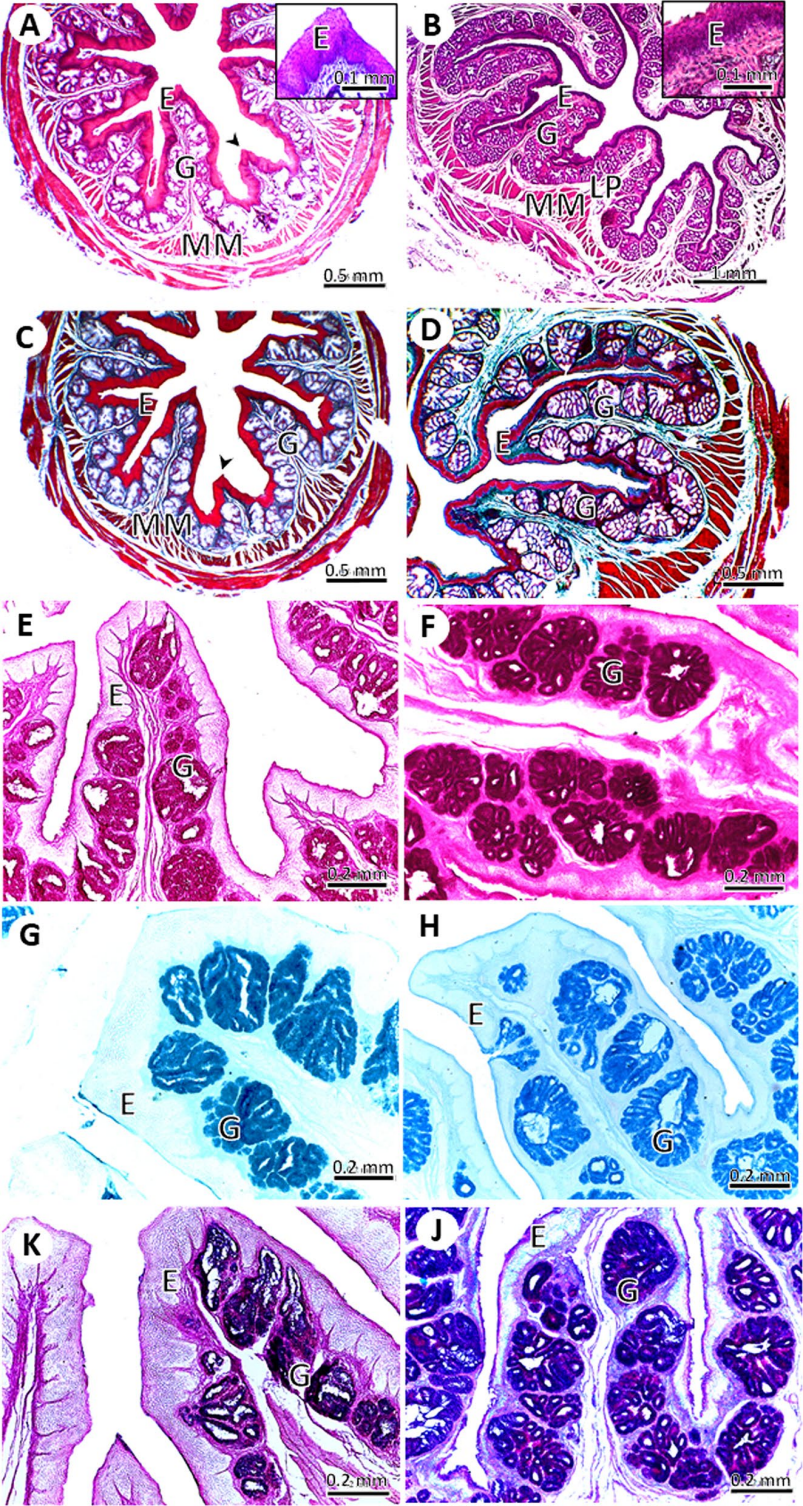
Light micrographs of the thoracic part of the esophagus in young (**A**, **C**, **E**, **G**, **I**) and adult (**B**, **D**, **F**, **H**, **J**) pigeons. The mucosal folds were lined by non-keratinized stratified squamous epithelium (**E**), lamina propria (LP), muscularis mucosa (MM), and gland (G). (**A**, **B**) stained with HE stain, (**C**, **D**) stained with Crossman’s trichrome stain, (**E**, **F**) stained with PAS technique, (**G**, **H**) stained with Alcian blue; and (**I**, **J**) stained with Alcian blue-PAS technique

The mucosa of the thoracic part of the esophagus formed several primary leaf-like mucosal folds and rarely showed secondary folds. The mucosal folds were significantly higher (1.55 ± 0.31 *Vs*. 0.86 ± 0.14 mm, *P* = 0.04) and in adult pigeons than in young pigeons. But no significant difference was present in the fold width between adult and young pigeons (0.79 ± 0.11 *Vs*. 0.47 ± 0.07 mm, *P* = 0.06). Both the primary and secondary folds contained mucous glands that secreted acid and neutral mucopolysaccharides. Only the primary folds contained smooth muscle at the base of the fold (Fig. 8).

## Discussion

In the present study, the esophagus of pigeons was divided into three parts: the cervical part, crop, and thoracic part similar to that reported in rock pigeons, rock doves, Eurasian collared doves, quails, house sparrows, ducks, and cattle egrets [6, 7, 15, 34–38]. In contrast, the esophagus of homing pigeons and partridges consists of two parts: cervical and thoracic parts [16, 21].

The current results showed that the cervical part of the esophagus was longer than the thoracic part, similar to other avian species, such as homing pigeons [21], quails [18], Eurasian common moorhens [39], partridges [16], and grey-backed shrikes [40]. In contrast, the cervical part of the esophagus in chicken [41] and Garganey [42], is shorter than the thoracic part.

The position of the cervical esophagus varied according to the region of the neck, being in the midline dorsal to the larynx in the cranial cervical region and inclined toward the right ventrolateral side of the neck in the middle cervical region. The displacement of the trachea on the side of the neck contralateral to the esophagus is due to the large, bilobed crop of the pigeon enables the passage of ingested food towards the large and bilobed crop at the thoracic inlet [43]. The cervical esophagus is a subcutaneous structure that can be palpated; therefore, it is easily accessible for surgery [9].

Similar to the present findings, the cervical esophagus of pigeons, quails, ducks, and cattle egrets is characterized by the presence of fine longitudinal folds [6, 34]. In contrast the cervical esophagus of homing pigeons [21], wood pigeons [14], rock doves [37], house sparrows [15], Eurasian common moorhen [39], and Garganey [42] have well-developed longitudinal folds. The differences between the size of the folds in these birds might be attributed to the different sizes of the swallowed food; birds that swallow bigger food items have deeper and wider folds than birds that swallow smaller food items [14]. Generally, the longitudinal folds of the esophagus facilitate its distensibility [34, 35].

In most birds, the cervical esophagus expands just before entering the thoracic cavity [8, 9], forming either a spindle-shaped, unilobed, or bilobed dilatation, the crop [44]. The size and shape of crop are species-specific characteristics, depending on their diet [10]. In agreement with the current findings, the crop of pigeons, doves, and rock doves is a large, bilobed non-glandular sac formed of two diverticula on either side of the esophagus [8, 12, 37]; therefore, it has a saccular appearance [45]. In chickens, the crop is a ventral diverticulum of the esophagus [9, 44]. However, in house sparrows, it is a bag-like structure [15]. In ducks and geese [8, 9], the crop is represented by a spindle-shape enlargement. However, the crop is absent in Garganeys, Eurasian common moorhens, grey-backed shrikes, owls, gulls, and penguins [9, 39, 40, 42]. For example, grey-backed shrikes tore their food on tree branches therefore, they do not need a crop to store food [40]. In Garganeys, the esophagus is thickened at the thoracic inlet to compensate for the absence of the crop [42].

The avian crop performs several functions, including temporary storage of the ingested food before passing to the proventriculus [10, 46] and softening of the swallowed grains by the action of salivary and esophageal gland secretions, water, amylase enzyme, and bacterial autolytic effect to facilitate grinding and enzymatic digestion of grains [47, 48]. In addition, some nutrients, such as glucose, β-carotene, and threonine, are absorbed directly from the crop [10]. In pigeons, doves, flamingos, and some penguins, the crop produces crop milk, a lipid-rich material regurgitated by the parents to feed their young, which is considered the only source of food during the first week after hatching [8, 46]. Crop milk is highly nutritious; it is rich in protein and fat, but poor in carbohydrates. It provides an easily digestible food that is rich in immunoglobulin A, and contains glycoproteins, transferrin, and pigeon milk growth factor [10]. Crop milk consists of desquamated mucosal epithelial cells lining the crop, and is mainly produced in the lateral diverticula [9, 12]. During the productive period, the walls of the diverticula thicken under the influence of prolactin hormone. Crop milk is strongly similar to mammalian milk, but is deficient in carbohydrates and calcium [12]. In hoatzin, the crop is considered the site of food digestion by microbes [46]. Furthermore, crops play an essential role in innate immunity as the first major defense against foodborne pathogens [49].

In agreement with the present findings, the crops of pigeons, doves, quails, sparrows, gallinaceous birds, and chickens have longitudinal folds on their inner surfaces. These folds make it distensible to accommodate large amounts of rapidly swallowed grains [15, 34, 44–48]. It is worth mentioning that the weight and volume of a young pigeon’s crop increases rapidly, approximately 10-fold, in the first postnatal week and remain constant afterwards. This rapid increase allows crops to hold larger amounts of ingested cereals mixed with crop milk [45].

In accordance to the current result, the thoracic esophagus of pigeons, rock doves, ducks, and cattle egrets has thicker longitudinal folds than those of the cervical esophagus [6, 37].

The lining epithelium of the cervical esophagus, crop, and thoracic esophagus of pigeons is composed of stratified squamous non-keratinized epithelium similar to that reported in pigeons, quails, cattle egrets, and ducks [6, 18, 22]. In contrast, stratified squamous keratinized epithelium is mainly found in the common rock and wood pigeons, rock doves, collared doves, domestic fowl, house sparrows, kestrels, and linnets [7, 14, 19, 32, 37]. The mucosal folds of the cervical esophagus were branched and consisted of primary, secondary, and tertiary folds. The shape of the mucosal folds differed among different bird species, being highly branched in pigeons, wave-like in rock pigeons, finger-like in cattle egrets and wood pigeons, and leaf-like in ducks [6, 7, 14]. Moreover, the muscularis mucosa was more well-developed in adults than in young pigeons. Peristaltic contraction of the inner circular and outer longitudinal muscles in the tunica muscularis pushes food caudally through the esophagus [11].

The mucosal folds of the thoracic esophagus were leaf-like, similar to those reported in pigeons [6, 14]. In this regard, the mucosal folds are finger-like in cattle egrets and orange leaf-like in ducks [6]. It is suggested that the variations in the shape of the mucosal folds are associated with the type and size of the food consumed by each bird species. Additionally, the shape and height of the mucosal folds, as well as the branching of the mucosal folds, differed between the young and adult pigeons. Moreover, the density of the gland lobules was higher in adult pigeons than in young pigeons. In agreement with the present results, the height of the mucosal folds increases with age in chickens [19].

The present SEM findings revealed difference in the distribution of the esophageal gland openings in the different parts of the esophagus of both adult and young pigeons, whereas the cervical part of the esophagus and the lateral diverticula were devoid of gland openings. The distribution of esophageal glands varies among different bird species. In agreement with the current results, esophageal glands are absent in the cervical esophagus of pigeons [6], rock pigeons [7], wood pigeons [14], Eurasian collared doves [36], and Japanese quails [35]. In contrast, the cervical esophagus of the Eurasian common moorhens [39] has several esophageal gland openings. Previous studies have reported the presence of several salivary glands in the oropharyngeal cavity of pigeons, including maxillary, palatine, sphenopterygoid, and lingual salivary glands [50, 51]. These glands produce a large amount of saliva, which lubricates the ingested dry seeds and facilitates swallowing [51] which is suggested to be a morphological adaptation in pigeons to compensate for the absence of esophageal glands in the cervical esophagus. However, crops of collared doves [32], chickens [44], cattle egrets, and ducks [22] contain glands. In contrast to the present results, the crops of domestic pigeons are devoid of glands [22].

Histochemical examination confirmed the SEM results and showed that the glands were mucous type. The type of esophageal gland also varied among different bird species. The glands are purely mucous in pigeons [6, 47], homing pigeons [21], rock doves [37], collared doves, house sparrows [32], quails [34], chickens [44, 48], cattle egrets, and ducks [22]. However, they are seromucous (mixed) in rock doves, collared doves, and rose-ringed parakeets [32]. The mucosal glands present in the thoracic esophagus facilitate the swallowing process by lubricating and moistening food [34]. The mucins secreted by the esophageal glands in the central part of the crop and thoracic esophagus serve as mucosal barriers [18]. The abundant and large mucous glands in pigeons produce a significant amount of mucus for wrapping the ingested grains and seeds, which might be an adaptation of pigeons to their granivorous diet [21].

## Conclusions

The present study showed variations in the structure of different parts of the esophagus in pigeons. These variations included the shape of the mucosal folds and distribution of the esophageal glands. Additionally, the present results revealed variations in the shape, height, and degree of branching of the mucosal folds and the density of the gland lobules between young and adult pigeons. The variations between young and adult pigeons suggest a functional adaptation of adult pigeons to their diet compared to young pigeons. Knowledge of the morphological structure of the normal esophagus and crop of pigeons will help properly interpret the histopathological changes induced by trichomonas infection in pigeons.

## Data Availability

The datasets used and/or analyzed during the current study are available from the corresponding author on reasonable request.
